# Selected Aspects of Using the Telemetry Method in Synthesis of RelNav System for Air Traffic Control

**DOI:** 10.3390/ijerph17010213

**Published:** 2019-12-27

**Authors:** Milan Džunda, Natália Kotianová, Peter Dzurovčin, Stanislav Szabo, Edina Jenčová, Iveta Vajdová, Peter Koščák, Dorota Liptáková, Peter Hanák

**Affiliations:** 1 Department of Air Transport Management, Faculty of Aeronautics, Technical University of Košice, Rampová 7, 04121 Košice, Slovakia; milan.dzunda@tuke.sk (M.D.); peter.dzurovcin@tuke.sk (P.D.); stanislav.szabo@tuke.sk (S.S.); iveta.vajdova@tuke.sk (I.V.); peter.koscak@tuke.sk (P.K.); dorota.liptakova@tuke.sk (D.L.); peter.hanak@tuke.sk (P.H.); 2 Transport Authority, 823 05 Bratislava, Slovakia; natalia.kotianova@nsat.sk

**Keywords:** air traffic management, accuracy, relative navigation, pseudo-range, position error

## Abstract

Accuracy is an important factor in air traffic management which is why high requirements are necessary for each navigation system. The aim of this article is to describe the principles of the RelNav system and telemetry and their accuracy. We present the algorithms of the relative navigation system, which could be used for air traffic control in the case of the unavailability of satellite navigation system signals. This article sums up the different positioning methods, and deals with the accuracy of the relative navigation system (RelNav). Furthermore, the article considers the factors that influence the positioning accuracy. For this task, a computer simulation was created to evaluate the accuracy of the telemetric method. Next, we discuss the principles of telemetry and algorithms for calculating the position of the flying object (FO).

## 1. Introduction

Nowadays, the position of a flying object (FO) is determined by satellite systems that use the telemetric method. In the near future, global navigation satellite systems will become the primary systems in air navigation while other alternative options are being developed and may be used as a backup in case of satellite outage. The RelNav system could be one of these alternative options. It determines the position of the FO by receiving messages from other users. We expect that the RelNav system will operate within the aviation communication network. The position of the FO is determined by geocentric coordinate systems in three-dimensional space. Satellite navigation systems are the primary means of future navigation and communication infrastructure. The status quo flows from the program NextGen (FAA) and the concept of ATM/CNS—Communication, Navigation and Surveillance Systems for Air Traffic Management (Eurocontrol). The proposed method is based on a relative navigation system in an aviation communication network. This system uses a method of measuring the time of arrival (TOA) of signals received from other users within an aviation communication network. Positioning in RelNav mode consists of measuring the distance between the source of transmission (SoT) with known coordinates and the unknown flying object by measuring the TOA [1,2]. Nowadays, relative navigation systems are used in military aviation and the algorithms of their operation are secret [3,4]. We have not performed further analytical analysis of the RelNav system as yet because we are not able to carry out an experiment with real measurements as the proposed RelNav system has not been designed yet. Therefore, we cannot make practical measurements with this system. The main aim of this paper was to verify the proposed algorithms of the RelNav system through simulation and the modeling process. The position of the FO that works in an aviation communication network can be determined by using different methods such as Doppler methods, the interferometric method, telemetry method, quadrant method, and combined methods. A common feature of all methods is that for positioning, they need to know the position of the other users working in the aviation communication network. The various methods differ from each other in the measured parameter of electromagnetic waves (transmitted by the signal). The proposed method of RelNav takes into account the position of other users (FO) in the aviation communication network and the time of arrival of signal transmitting from these users. The proposed method is based on pseudo range modeling, where the pseudo range is the distance between the users and is calculated with time errors because of the synchronization problem. TOA is the time of arrival of the signal that is received by an unknown user and is measured according to the user’s time. Tasks of the synthesis and analysis navigation systems are based on the application of mathematical models of dynamic systems (object of navigation), signals, interference, and information processes [5,6,7].

## 2. Telemetry and Algorithms for Calculating the Flying Object Position

Telemetry is the automatic measurement and wireless transmission of data from remote sources. The location of the FO is determined by acquiring the transmissions from three (or more) different locations to triangulate the location of the device. Each user in communication network transmits a signal that contains information about its position and the current time at regular intervals. These signals, travelling at the speed of light, are intercepted by a receiver of an unknown user, which calculates how far each user is based and how long it takes for the signal to arrive. The range from the other users is determined by the time the signal is received. To ensure the work of the RelNav system, at least three users must be visible to the unknown receiver at all times. The principle of relative navigation is described in detail in [2,3]. The location of the unknown user is calculated by the following solution system of three equations [8,9]:

(1) $$\sqrt{{\left(x_{i}-x\right)}^{2}+{\left(y_{i}-y\right)}^{2}+{\left(z_{i}-z\right)}^{2}}=d_{i}$$

where *x_i_*, *y_i_*, *z_i_* is the geocentric coordination of the user; *d_i_* is the range between an unknown receiver and each transmitter; and *x*, *y*, *z* is the geocentric coordination of an unknown receiver.

The distances to the users are derived from the transmitted signals and are affected by uncertainties in clock setting, therefore they are normally referred to as pseudo-ranges. There is a time synchronization problem of each user’s clock. The pseudo-range measurements lead to a pseudo-ranging four point problem, which is the problem of determining the four unknowns. The unknowns comprise the three components of the receiver position {X, Y, Z} and the stationary receiver range bias. Four pseudo-range equations are expressed algebraically as [10]:

(2) $${\left(x-x_{1}\right)}^{2}+{\left(y-y_{1}\right)}^{2}+{\left(z-z_{1}\right)}^{2}-{\left(b-d_{1}\right)}^{2}=0$$

(3) $${\left(x-x_{2}\right)}^{2}+{\left(y-y_{2}\right)}^{2}+{\left(z-z_{2}\right)}^{2}-{\left(b-d_{2}\right)}^{2}=0$$

(4) $$\;{\left(x-x_{3}\right)}^{2}+{\left(y-y_{3}\right)}^{2}+{\left(z-z_{3}\right)}^{2}-{\left(b-d_{3}\right)}^{2}=0$$

(5) $$\;{\left(x-x_{4}\right)}^{2}+{\left(y-y_{4}\right)}^{2}+{\left(z-z_{4}\right)}^{2}-{\left(b-d_{4}\right)}^{2}=0$$

where *d*_1_, *d*_2_, *d*_3_, *d*_4_ are the measured pseudo-distances from a source of transmission to receivers; *x*_1–4_, *y*_1–4_, *z*_1–4_ are the coordinates of the users; *x*, *y*, *z* are the coordinates of the receiver; and *b* is the shift of the time basis of the receiver converted into distance.

For subtraction of Equation (5) from Equation (2), is valid:

(6) $$f_{14}=x_{14}x+y_{14}y+z_{14}z+d_{41}b+e_{14}$$

For subtraction of Equation (5) from Equation (3), is valid it holds:

(7) $$f_{24}=x_{24}x+y_{24}y+z_{24}z+d_{42}b+e_{24}$$

For subtraction of Equation (5) from Equation (4), is valid:

(8) $$f_{34}=x_{34}x+y_{34}y+z_{34}z+d_{43}b+e_{34}$$

If variable *b* is considered as constant (the so-called factor of homogenization), then, depending on Equations (6)–(8), a system of three equations with three unknown variables is obtained. We applied the method of resultants of the multi-polynomials to solve the system of linear equations where *x = g(b)*, *y = g(b)*, *z = g(b)* [3,7,8].

The following is valid:

(9) $$f_{1}=\left(x_{14}x+d_{41}b+e_{14}\right)k+y_{14}y+z_{14}z$$

(10) $$f_{2}=\left(x_{24}x+d_{42}b+e_{24}\right)k+y_{24}y+z_{24}z$$

(11) $$f_{3}=\left(x_{34}x+d_{43}b+e_{34}\right)k+y_{34}y+z_{34}z\;$$

If the variable “*turns*” into the factor of homogenization, Jacobi’s determinant of the coordinate *x* by Equations (9)–(11) is expressed as:

(12) $$J_{x}=det\left(\begin{array}{cc} \frac{df_{1}}{\begin{array}{c} dy\\ \frac{df_{2}}{dy} \end{array}} & \frac{df_{1}}{\begin{array}{c} dz\\ \frac{df_{2}}{dz} \end{array}}\\ \frac{df_{3}}{dy} & \frac{df_{3}}{dz} \end{array}\begin{array}{cc} \frac{df_{1}}{\begin{array}{c} dk\\ \frac{df_{2}}{dk} \end{array}} & \\ \frac{df_{3}}{dk} & \end{array}\right)=\;\det$$

In compliance with Equations (9)–(11), for *y* = *g (b)* is valid:

(13) $$f_{4}=\left(y_{14}y+d_{41}b+e_{14}\right)k+x_{14}x+z_{14}z$$

(14) $$f_{5}=\left(y_{24}y+d_{42}b+e_{24}\right)k+x_{24}x+z_{24}z$$

(15) $$f_{6}=\left(y_{34}y+d_{43}b+e_{34}\right)k+x_{34}x+z_{34}z$$

Jacobi’s determinant for the coordinate *y* is expressed as:

(16) $$J_{y}=det\left(\begin{array}{cc} \frac{df_{4}}{\begin{array}{c} dx\\ \frac{df_{5}}{dx} \end{array}} & \frac{df_{4}}{\begin{array}{c} dz\\ \frac{df_{5}}{dz} \end{array}}\\ \frac{df_{6}}{dx} & \frac{df_{6}}{dz} \end{array}\begin{array}{cc} \frac{df_{4}}{\begin{array}{c} dk\\ \frac{df_{5}}{dk} \end{array}} & \\ \frac{df_{6}}{dk} & \end{array}\right)=det$$

In compliance with Equations (9)–(11), for *z* = *g (b)* is valid:

(17) $$f_{7}=\left(z_{14}z+d_{41}b+e_{14}\right)k+x_{14}x+y_{14}y$$

(18) $$f_{8}=\left(z_{24}z+d_{42}b+e_{24}\right)k+x_{24}x+y_{24}y\;$$

(19) $$f_{9}=\left(z_{34}z+d_{43}b+e_{34}\right)k+x_{34}x+y_{34}y$$

Jacobi’s determinant for coordinate *z* is expressed as:

(20) $$J_{z}=det\left(\begin{array}{cc} \frac{df_{7}}{\begin{array}{c} dx\\ \frac{df_{8}}{dx} \end{array}} & \frac{df_{7}}{\begin{array}{c} dy\\ \frac{df_{8}}{dy} \end{array}}\\ \frac{df_{9}}{dx} & \frac{df_{9}}{dy} \end{array}\begin{array}{cc} \frac{df_{7}}{\begin{array}{c} dk\\ \frac{df_{8}}{dk} \end{array}} & \\ \frac{df_{9}}{dk} & \end{array}\right)=det$$

From Equations (12), (16), and (20), we obtained the value of the determinants *Jx*, *Jy*, *Jz*. We obtained the variable equations *x = g(b)*, *y = g(b)*, *z = g(b)* by applying the value of each determinant. Substituting the expression for *x*, *y*, *z* into Equation (2), the quadratic function for the unknown variable *b* is obtained:

(21) $$Ab^{2}+Bb^{2}+C=0$$

**Figure 1:**
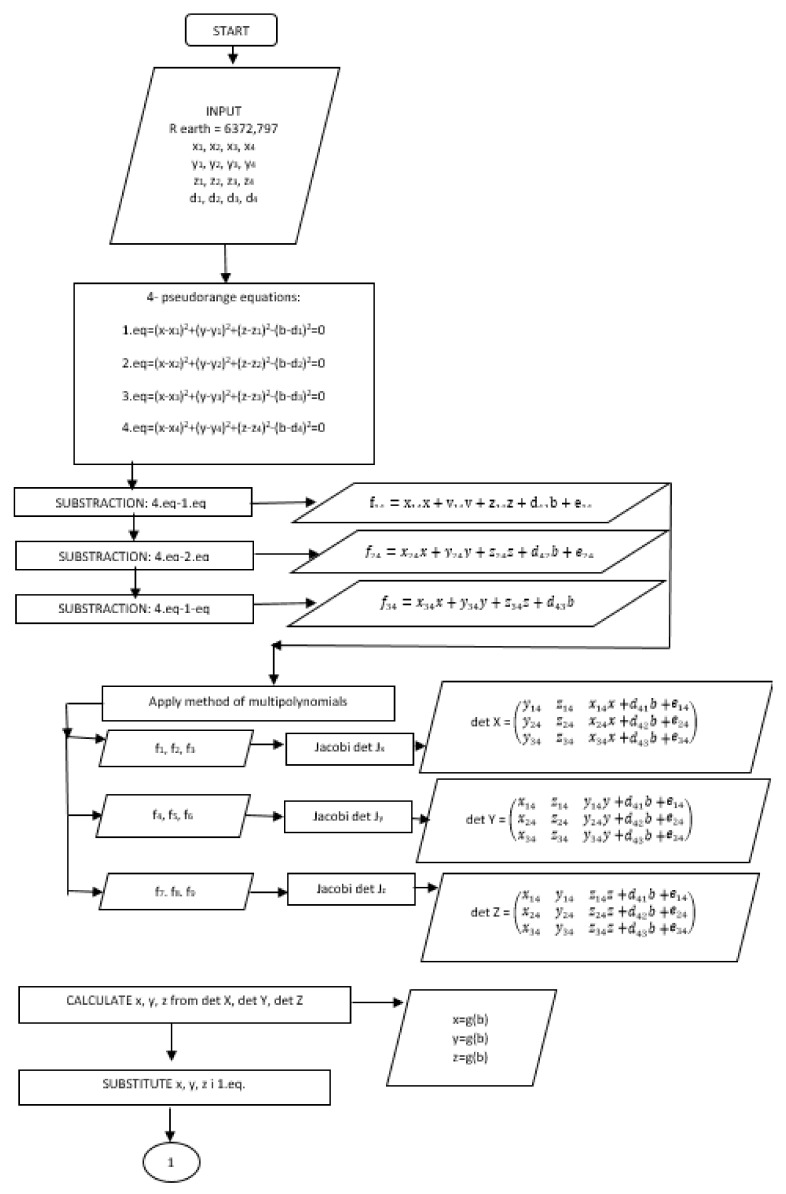

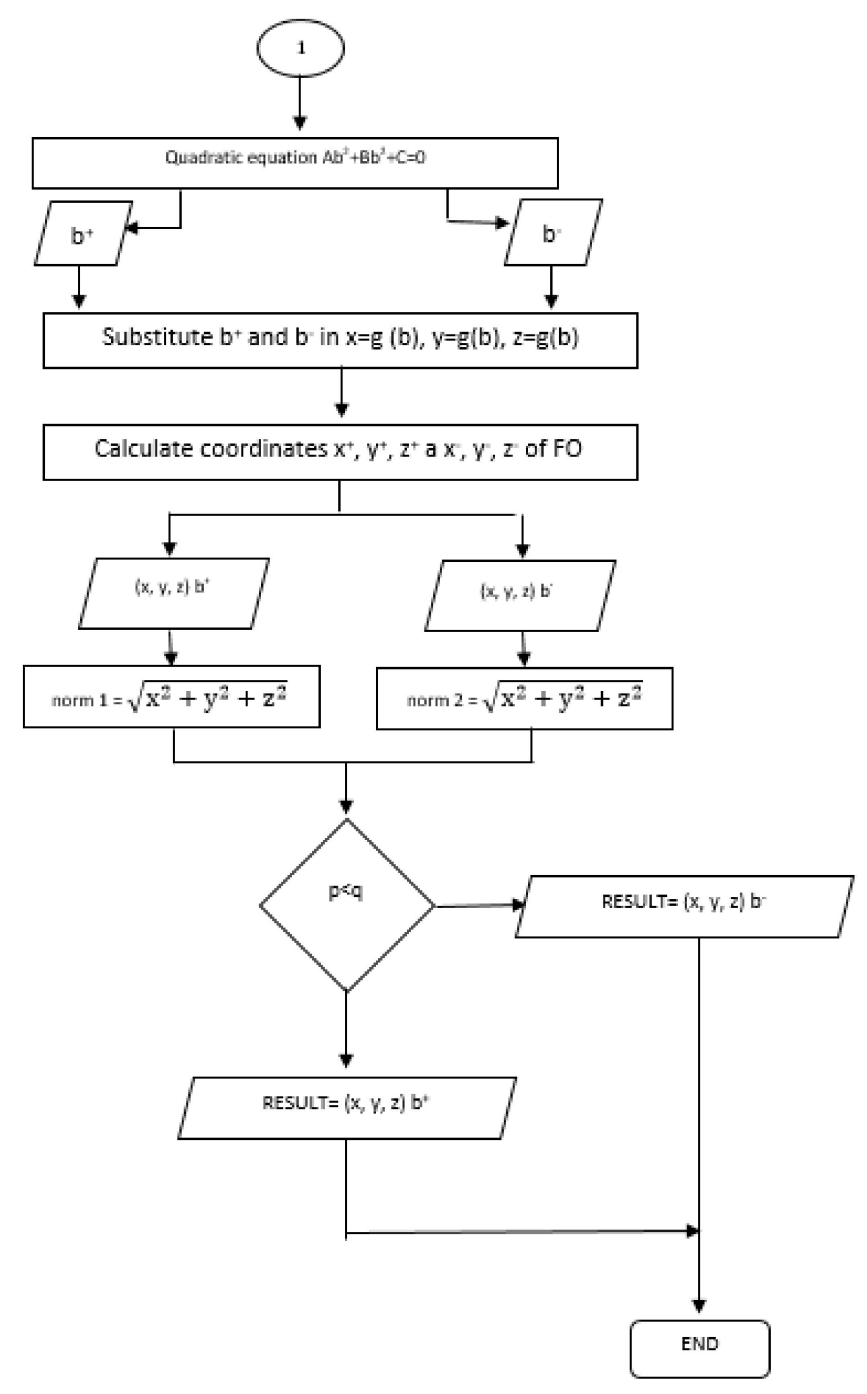
The flowchart of the proposed algorithm.

Solutions of the quadratic Equation (21) have two roots, b+ and b−. Therefore, we have to calculate the coordinates of the FO (x+, y+, z+) and P (x−, y−, z−). To come to the correct solution, we have to calculate the norm ($\mathrm{norm}=\sqrt{x^{2}+y^{2}+z^{2}}$) for (x, y, z) b− and (x, y, z) b+. If the coordinates of the receiver are fed into the coordinate reference system, the norm of the position vector will be close to the value of the radius of the Earth (R_z_ = 6372.797 km) and this solution for the FO (x, y, z) will be regarded as a correct one [3,10,11]. The flowchart of the proposed algorithm is shown in Figure 1.

## 3. Modeling and Simulation of the RelNav System with Ideal Time Synchronization

We decided to conduct the computer simulation in MATLAB to analyze and evaluate the accuracy of the RelNav system operating in an aviation communication network. Computer simulation allowed us to save a lot of money in air transport [9,10]. We created the algorithms for solving the four pseudo-range Equations (2)–(5). By modeling the movement of the FO in 3D space, we set geographic coordinates of the FO. Geographic coordinates were subsequently transformed to the geocentric coordinate system in which the position was determined. The initial position of FO in space corresponds to real air traffic over the territory of the Slovak Republic. The initial conditions of each FO are shown in Table 1.

**Figure 2:**
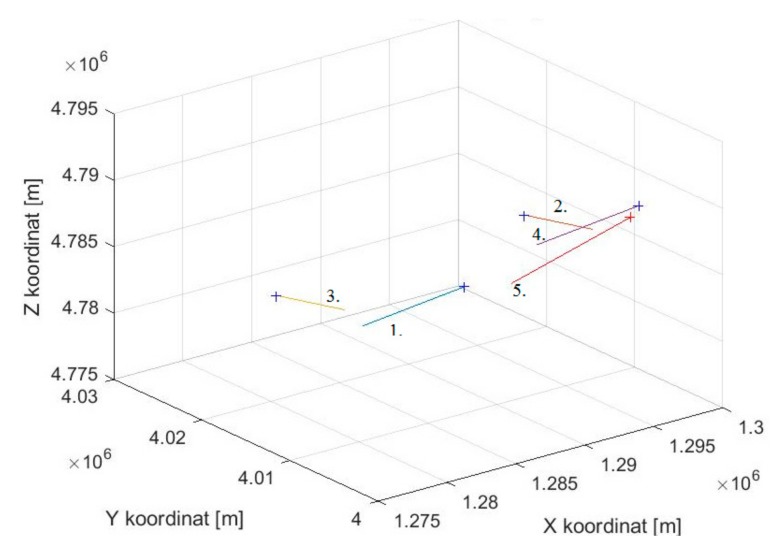
Simulation constellation of users in space.

Figure 2 shows the model of geometric constellation of five users in the communication network that are flying at the straight flight path.

**Figure 3:**
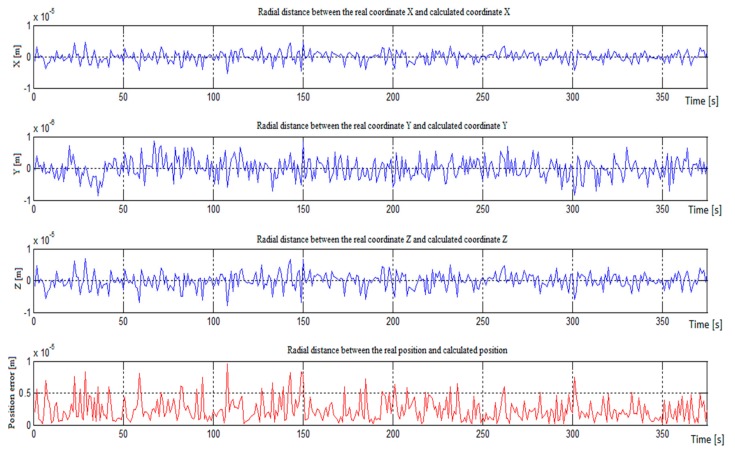
Positioning error in the case of ideal time synchronization.

We assumed that for these five users of the communication network, we knew the location of four FOs and wanted to determine the location of an unknown FO, whose trajectory is marked with the number 5. The results of the simulation of the determination of the location of the unknown FO (number five) are shown in Figure 3. Figure 3 shows the error of coordinates x, y, and z in meters and the error of the radial distance between the real position and calculated position. In the case of ideal time synchronization between the unknown receiver and all transmitters in the communication network, the determination positioning accuracy is in the range of 1 × 10^−6^ to 1 × 10^−5^ m.

If the ideal synchronization is achieved and no other signal noise occurs, the position of the FO can be calculated with maximum accuracy. However, it is very complicated to achieve the ideal time synchronization between all users. The ideal time synchronization can be achieved by transmitting the synchronization signal to all users or by equipping the aircraft with an accurate atomic clock [12]. In our research, we verified the impact of the geometry of the users of the communication network and their mutual synchronization on the accuracy of the determined location of an unknown object, which worked in this network.

## 4. Discussion

**Figure 4:**
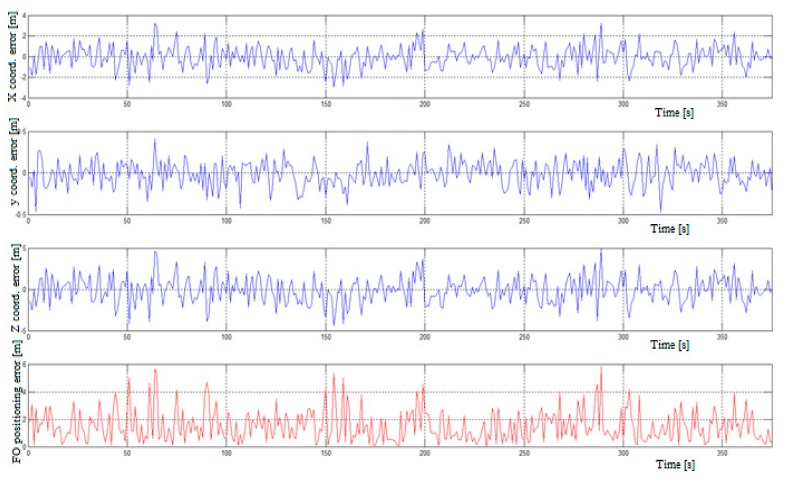
FO positioning error if k = 0.1 and D = 10.0 km.

The ideal time synchronization does not exist in real air traffic. Therefore, the accuracy is affected by a clock bias. In the next simulation, we considered the existence of a random error in the transmission time of signal transmitting from a single transmitter to a remote single receiver. Errors were derived from the random number generator with Gaussian distribution. The random numbers were in the range of (0, 1) and presents an error in coordinates X, Y, Z for each of the four users. These random errors were added to real coordinates that were derived from the model. In this way, we obtained a pseudo-range instead of the real range between users and analyzed how the pseudo-range affected the accuracy of positioning. One model situation involved all users flying on the straight path (Figure 2) and were spaced apart for 10 to 200 km gradually. The initial conditions of flight were the same as in the first simulation with ideal time synchronization (Table 1). Changing the constant k represents the value of a pseudo-range error. If the constant is greater, the positioning error is also greater. Figure 4 shows the positioning errors for the FO and the errors of coordinates X, Y and Z for the distance between FO D = 10.0 km and the constant k = 0.1. The graph shows the error of the x, y, and z coordinates and the FO positioning error in meters. In the case of a time synchronization error between an unknown FO receiver by all transmitters in the communication network (k = 0.1), the positioning error was in the range of 0 to 7 m. The mean value of the radial distance positioning error (ErrP) between the real and calculated position is presented in Table 2. The ErrP when changing the distance between the FO from 10 to 200 km can rise by up to 500 m, which corresponds to a time error of about 1.67 μs. This indicates importance of the time synchronization of the communication network. 

**Figure 5:**
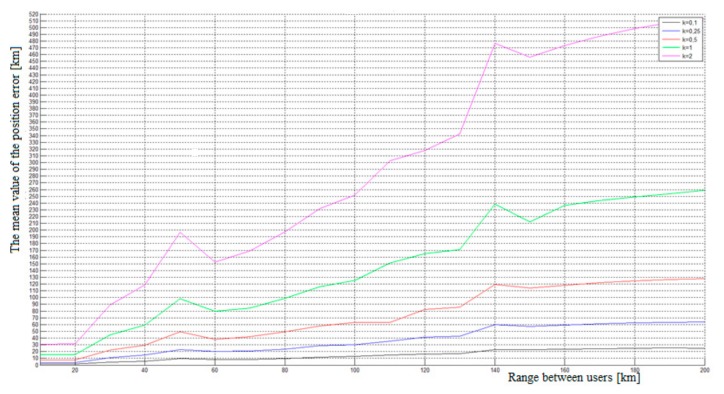
The comparison of position errors for various ranges of the pseudo-range error.

We created a simulation with the constants 0.1, 0.25, 0.5, 1, and 2 to analyze the influence of different pseudo-range errors to determine the position. We also analyzed the influence of the distance between the unknown FO and the users of the FO 1 to FO 4 network, which we labeled D (see Figure 5). Figure 5 shows the mean values of the radial distance between the real and calculated position ErrP [m] for different values of constant k and distance D. The simulation results show that the position error increases with increasing distance between users. In the case of using the constant k = 0.1, the ErrP is maintained under the value 30 m, and represents the time error 1 × 10^−7^ s. If the random errors come from the greater range, the simulation has the same progress but with a higher value of position errors.

The graphic comparison of position errors is shown in Figure 4 and depends on the selected constants. The graph shows that the greater the pseudo-range error and distance, the greater the position error ErrP. Therefore, we can assume that this method is not suitable for a user that is at a remote location more than 40 km from any source of signal transmission. Sufficient accuracy could be achieved when the signals were transmitted in a radius of 40 km. Therefore, the receiver should consist of algorithms that recognize the intensity of the incoming signals and select the strongest one to calculate their own position.

## 5. Conclusions

The paper deals with the assessment of the accuracy of the RelNav system. The submitted contribution discusses the principle of telemetry as a method of determining the positioning of the FO and presents two simulation cases. The first considers the situation with the ideal time synchronization in the communication network and no errors in the transmission of information. The simulation results showed that in the case of the ideal time synchronization between the unknown receiver and all transmitters in the communication network, the determination of the positioning accuracy was in the range of 1 × 10^−6^ to 1 × 10^−5^ m. In practice, such a high determination of the positioning accuracy of an unknown FO (FO 5) cannot be achieved because we are unable to ensure the synchronization of the communication network of users and the precise determination of the coordinates of known FOs (FO 1 to 4). The second simulation takes into account the existence of an error in the synchronization of the communication network of users and inaccuracy in the determination of the FO coordinates (FO 1 to 4), which we consider to be known. We also investigated how the geometry of the communication network users (FO 1 to 4) affected the accuracy of determining the position of the unknown FO 5.

The requirements for accuracy in air traffic are very strict. The results of the simulation showed that the accuracy of the position determination of the FO operating in the aviation communication network depends on the synchronization of the network and on the geometry of the network users. Table 2 shows that the errors in determining positioning FO 5 varied from 1.5 to 513 m, depending on the accuracy of the network synchronization (k = 0.1 to 2) and the geometry of network users (D = 10 to 200 m).

The accuracy of this system was worse than accuracy of the current GNNS. The RelNav system could be used in cases of GNNS outage for FO navigation up to a distance of 70 km while ensuring the required synchronization of the communication network. However, without good time synchronization, it is only useful for a short spacing between flight objects. Therefore, the RelNav system can be used among other approaches for flights in formation or during in-flight refueling. The telemetry method could be used to design anti-collision systems and navigation systems, which increases air traffic safety.

The accuracy of the relative navigation system has been assessed, for example, in [13,14]. It is stated that the accuracy of relative navigation can be increased using the extended Kalman filter. In this case, the authors give the accuracy of about one meter. This consideration goes beyond the use of the telemetry method to determine the position of flying objects in the relative navigation system. The telemetry method of positioning is also used in satellite navigation systems. The positioning accuracy of these systems is from one to up to tens of meters and depends on the quality of the available measurement signals and the intensity of the interference.

## Figures and Tables

**Table 1:** The initial coordinates of flying objects acquired from the model.

	Ellipsoidal Height [km]	X [km]	Y [km]	Z [km]
**1. FO**	10	4020.605594865663	1286.566147141365	4778.506510936486
**2. FO**	10.351	4007.782065714437	1295.413452281405	4787.291580824662
**3. FO**	9.302	4016.257242495344	1282.749739363568	4782.227863636937
**4. FO**	8.953	4006.592544950288	1290.575848954818	4787.722024180670
**5. FO**	9.654	4013.608340680915	1293.310189580862	4782.079490092985

**Table 2:** The results from the simulation with the pseudo-range error.

	Mean Value of Radial Distance between the Real and Calculated Position ErrP [m]				
Distance [km]	K = 0.1	K = 0.25	K = 0.5	K = 1	K = 2
**10**	1.52665	3.52231	7.63329	15.26657	30.53310
**30**	4.45322	11.13307	22.26615	44.53233	89.06476
**50**	9.82508	22.76620	49.12542	98.25086	196.50177
**70**	8.46138	21.15344	42.30688	84.61375	169.22738
**90**	11.60177	29.00443	58.00886	116.01767	232.03515
**110**	15.14273	35.68129	62.98029	151.42725	302.85429
**130**	17.11375	42.78437	85.56873	171.13738	342.27442
**150**	22.79639	56.99096	113.98189	211.92773	455.92653
**170**	24.36820	60.92050	121.84096	243.68171	487.36260
**190**	25.35758	63.39393	126.78781	253.57543	507.15006
**200**	24.75452	64.13355	128.26705	258.72705	513.06684
